# Effectiveness of dual-site transcranial magnetic stimulation on motor function and activities of daily living in stroke patients: a systematic review and meta-analysis of randomized controlled trials

**DOI:** 10.3389/fneur.2025.1630876

**Published:** 2025-07-14

**Authors:** Jiawei Qin, Zhenzhen Hong, Jingfeng Wang, Yi Zhang, Huihong Zhuang, Shanshan Hong, Liling Zheng

**Affiliations:** ^1^Department of Rehabilitation Medicine, Quanzhou First Hospital Affiliated to Fujian Medical University, Quanzhou, China; ^2^Department of Endocrinology, Quanzhou First Hospital Affiliated to Fujian Medical University, Quanzhou, China; ^3^Department of Obstetrics and Gynecology, Quanzhou Women’s and Children’s Hospital, Quanzhou, China; ^4^Department of Cardiovascular Surgery, Quanzhou First Hospital Affiliated to Fujian Medical University, Quanzhou, China

**Keywords:** transcranial magnetic stimulation, motor function, activities of daily living, stroke, systematic review

## Abstract

**Background:**

Dual-site transcranial magnetic stimulation (TMS) has emerged as a promising neuromodulation technique in stroke rehabilitation. By targeting multiple brain regions, dual-site TMS may enhance neuroplasticity more effectively than single-site stimulation. However, its clinical efficacy remains uncertain.

**Objective:**

To systematically evaluate the effects of dual-site TMS in improving motor function and activities of daily living (ADL) in patients with stroke.

**Methods:**

We conducted a systematic review and meta-analysis of randomized controlled trials (RCTs) following PRISMA guidelines. Seven electronic databases were searched from inception to February 19, 2024. Studies comparing dual-site TMS with single-site TMS, sham dual-site TMS, or routine rehabilitation in stroke patients were included. Outcomes included Fugl-Meyer Assessment (FMA), FMA-Upper Limb (FMA-UL), Action Research Arm Test (ARAT), Barthel Index (BI), Modified Barthel Index (MBI), Wolf Motor Function Test (WMFT), and others. Methodological quality was assessed using the PEDro scale. Meta-analyses were performed using a random-effects model.

**Results:**

Fourteen RCTs involving 724 participants were included. Dual-site TMS significantly improved upper limb motor function compared with single-site TMS (MD = 7.07, 95% CI: 1.46 to 12.68, *p* < 0.001) and sham dual-site TMS (MD = 14.45, 95% CI: 6.23 to 22.66, *p* < 0.001). ADL outcomes also favored dual-site TMS over single-site TMS (MD = 9.90, 95% CI: 7.82 to 11.98, *p* < 0.001) and sham dual-site TMS (MD = 21.13, 95% CI: 9.37 to 32.88, *p* < 0.001). Subgroup analyses suggested enhanced benefits in subacute phase stroke and in protocols with >20 sessions. Sensitivity analysis confirmed robustness of findings. No serious adverse events were reported.

**Conclusion:**

Dual-site TMS combined with routine rehabilitation is more effective than single-site TMS or sham dual-site TMS in improving motor function and ADL among stroke patients. These findings support its clinical application as an adjunct to conventional therapy. Further high-quality trials are needed to optimize stimulation protocols and confirm long-term effects.

## Introduction

Stroke remains one of the leading causes of mortality and long-term disability globally, according to the systematic analysis for the Global Burden of Disease Study 2021, there were 93·8 million stroke survivors, 11·9 million new stroke events, and 160·5 million disability-adjusted life-years (DALYs) from stroke, comprising 5·6% of all DALYs from all causes, the fourth leading cause of DALYs (1). Motor deficits, including hemiparesis and impaired coordination, affect approximately 80% of stroke survivors, severely limiting upper and lower limb function and diminishing independence in activities of daily living (ADL) (2). These deficits contribute to reduced quality of life, increased caregiver burden, and substantial socioeconomic costs, underscoring the urgent need for effective rehabilitation strategies (3, 4).

Conventional interventions, such as physical therapy, constraint-induced movement therapy, and robotic-assisted training, aim to promote neuroplasticity and functional recovery (5). However, their efficacy is often modest, particularly in patients with severe motor impairment or chronic-phase stroke, due to limited neural reorganization capacity and suboptimal engagement of residual neural networks (6). Non-invasive brain stimulation techniques, notably transcranial magnetic stimulation (TMS), have emerged as promising adjuncts to traditional therapies (7). By modulating cortical excitability and enhancing synaptic plasticity, TMS can facilitate motor recovery through mechanisms such as long-term potentiation (LTP), interhemispheric inhibition rebalancing, and activation of diaschisis-related pathways (8). High-frequency (≥5 Hz) rTMS (repetitive Transcranial Magnetic Stimulation) applied to the ipsilesional motor cortex (M1) or low-frequency (≤1 Hz) rTMS targeting the contralesional M1 has shown moderate benefits in improving limb function (9). In addition, intermittent Theta Burst Stimulation (iTBS) and continuous Theta Burst Stimulation (cTBS) show positive effect in improving motor function outcomes for stroke patients (10, 11). Notably, cerebellar TMS interventions have also demonstrated efficacy in enhancing post-stroke balance control and ADL (12), further expanding the clinical applicability of TMS strategies in stroke rehabilitation.

Despite these advances, single-site TMS protocols exhibit variable clinical outcomes, likely due to their inability to address the complex, distributed neural networks disrupted by stroke (13, 14). Motor recovery relies not only on local cortical excitability but also on interregional connectivity between motor, premotor, and cerebellar regions (15). Dual-site TMS, which simultaneously or sequentially stimulates two distinct brain targets (e.g., bilateral M1, or M1-cerebellum), may amplify therapeutic effects by synchronizing neural oscillations, enhancing cross-hemispheric communication, and promoting network-level reorganization (16). Motor recovery after stroke relies on the brain’s capacity for plasticity, and non-invasive brain stimulation (NIBS) methods such as rTMS, have shown promise in enhancing this plasticity by modulating cortical excitability (17, 18). Preclinical studies suggest that dual-site stimulation may induce stronger and more durable neuroplastic changes compared to single-site protocols, potentially by co-activating complementary pathways involved in motor planning and execution (19). Dual-site TMS has emerged as a promising non-invasive therapeutic intervention in stroke rehabilitation.

Current evidence on dual-site TMS in stroke rehabilitation remains fragmented. Some randomized trials report superior motor and ADL outcomes with dual-site versus single-site TMS or sham stimulation (20–23), however, significant variability in study designs, intervention parameters, patient characteristics, and outcome measures has made it challenging to reach a consensus on its overall effectiveness in stroke rehabilitation. A systematic synthesis of existing data is critical to clarify the efficacy of dual-site TMS, identify optimal protocols, and inform clinical recommendations. This meta-analysis aims to address three key questions: (1) Does dual-site TMS yield greater improvements in motor function and ADL than single-site TMS? (2) Is dual-site TMS more effective than sham stimulation? (3) What factors may moderate treatment effects? By integrating findings from recent high-quality trials, this study will provide evidence-based recommendations to optimize TMS protocols, bridge translational gaps, and advance personalized neurorehabilitation strategies for stroke survivors.

## Method

This systematic review and meta-analysis was conducted according to the Preferred Reporting Items for Systematic Reviews and Meta-Analysis (PRISMA) statement.

### Search strategy

A comprehensive literature search was performed in seven electronic databases, including PubMed, Embase, Cochrane Library (CENTRAL), Web of Science, China National Knowledge Infrastructure (CNKI), Wanfang and Chinese Scientific Journal (VIP) from their inception to February 19, 2024. The following search items combined Medical Subject Headings and key words to identify appropriate studies: (“transcranial magnetic stimulation” or “magnetic stimulation transcranial” or “stimulation transcranial magnetic” or “theta burst stimulation” or “iTBS” or “cTBS” or “TMS” or “rTMS”) AND (“Stroke” or “cerebrovascular accident” or “CVA” or “cerebrovascular apoplexy” or “brain vascular accident” or “cerebrovascular stroke” or “cerebral stroke” or “cerebrovascular accident”). The full search strategy in PubMed database was available in Supplementary Table 1. The reference of all included studies were manually screened to identify any missed eligible study. Endnote X9 (Thomson Reuters) was used to manage all references.

### Inclusion and exclusion criteria

Inclusion criteria were: ① target population: stroke survivor; ② interventions: any type of dual-site transcranial magnetic stimulation, including single-pulse TMS, rTMS, cTBS, and iTBS; ③ comparisons: dual-site TMS vs. single-site TMS/sham dual-site TMS/non-treatment, dual-site TMS + routine rehabilitation vs. single-site TMS + routine rehabilitation, dual-site TMS + routine rehabilitation vs. sham dual-site TMS + routine rehabilitation, dual-site TMS + routine rehabilitation vs. routine rehabilitation; ④ outcomes: at least one of motor function and activity of daily life measurements, such as Fugl-Meyer Assessment (FMA), Fugl-Meyer Assessment-upper limb (FMA-UL), Fugl-Meyer Assessment-lower limb (FMA-LL), Action Research Arm Test (ARAT), Ashworth or Modified Ashworth Scale (MAS), Wolf Motor Function Test (WMFT), modified Rankin Scale (mRS), Berg Balance Scale (BBS), Barthel Index (BI), Modified Barthel Index (MBI), and other motor function and ADL related outcomes; ⑤ study design: randomized controlled trials. Exclusion criteria were: ① animal model; ② repeated publications; ③ case reports, review, protocol, conference abstract, and letters to editor; ④ central-peripheral paired associative stimulation; ⑤ paired associated stimulation; ⑥ did not report motor function or ADL related outcomes.

### Data extraction

Duplicate references were removed using EndNote X9. Two independent reviewers screened the titles and abstracts of the retrieved studies to exclude irrelevant records. Full texts of potentially eligible articles were then reviewed in detail to confirm inclusion. Relevant data were extracted into a standardized form, including study authors, year of publication, participant characteristics (sample size, age, sex, and stroke duration), stroke type, intervention details, outcome measures, and adverse events. Discrepancies were resolved through discussion with a third reviewer. When essential data were missing or unclear, study authors were contacted. If results were available only in graphical format and could not be obtained from the authors, values were estimated using GetData Graph Digitizer version 2.25.^1^

### Risk of bias assessment

Methodological quality of the included studies was independently assessed by two reviewers using the Physiotherapy Evidence Database (PEDro) scale. The scale comprises 11 items evaluating aspects such as randomization, allocation concealment, baseline comparability, blinding (participants, therapists, assessors), follow-up adequacy, intention-to-treat analysis, between-group comparisons, and reporting of point estimates and variability. Each item was scored as 1 (criterion met) or 0 (criterion not met), yielding a maximum score of 10 (the first item is not included in the total score). Studies scoring ≥6 were considered high quality, scores of 4–5 were deemed moderate quality, and scores <4 were classified as low quality. Discrepancies were resolved through discussion with a third reviewer.

### GRADE assessment

We used the GRADE (Grading of Recommendations Assessment, Development and Evaluation) approach to assess the certainty of evidence across studies for each outcome (24). The domains considered included risk of bias, inconsistency, indirectness, imprecision, and publication bias. The certainty ratings were categorized as high, moderate, low, or very low. These evaluations were independently conducted by two authors, and discrepancies were resolved by consensus.

### Statistical analysis

For studies employing the same outcome measurement scale, meta-analyses were performed using Review Manager (RevMan) version 5.3. Continuous outcomes were summarized using the weighted mean difference (WMD) and corresponding 95% confidence intervals (CIs). The number of participants, post-intervention means, and standard deviations (SDs) in both experimental and control groups were extracted and analyzed. A random-effects model was applied given the expected heterogeneity among studies. Statistical significance was defined as a two-sided *p* < 0.05. Heterogeneity was assessed using the I^2^ statistic, with values interpreted as follows: low (<25%), moderate (25–50%), and high (>50%) (25). Subgroup analyses were conducted based on stroke phase (acute, subacute vs. chronic) and the total number of transcranial magnetic stimulation (TMS) sessions (≤10 vs. > 10 sessions). When meta-analyses included 10 or more studies, publication bias was evaluated using funnel plot asymmetry. In trials involving more than two groups with a shared control group, the shared group was evenly divided to prevent sample size inflation, allowing for independent pairwise comparisons. Sensitivity analyses were conducted by sequentially excluding individual studies to assess the robustness of the pooled estimates and explore potential sources of heterogeneity. Separate meta-analyses were conducted for each outcome measure when multiple instruments were used across studies. For outcomes reported in only a single study, results were summarized narratively rather than quantitatively. For studies reporting only mean values and 95% CIs, SDs were estimated by dividing the CI width by 3.92 and multiplying by the square root of the sample size. When only medians and interquartile ranges (IQRs) or ranges were reported, the means and SDs were approximated using established statistical methods depending on the data distribution and sample size (26).

## Result

### Study selection

**Figure 1:**
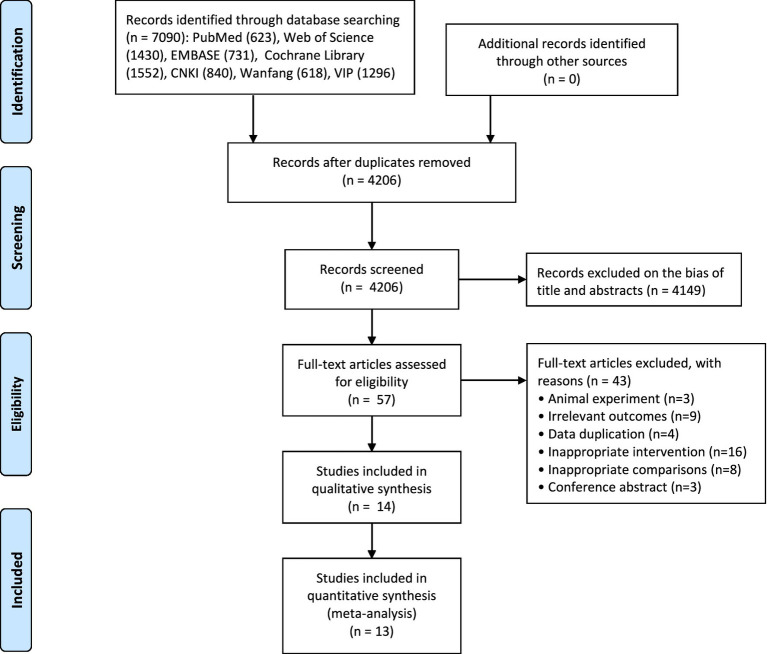
Flow diagram for studies selection.

A total of 7,090 articles were retrieved from the database search, 57 of which were retained after removing duplicates and irrelevant records. During the detailed full-text screening, 14 studies (20–23, 27–36) were excluded for not meeting the inclusion criteria with a total of 724 participants. The detailed search and selection process were presented in the flow diagram (Figure 1).

### Characteristics of the included studies

A total of 14 studies were included in the qualitative synthesis. The 14 included trials were published in 2013 (22), 2018 (20, 32), 2020 (36), 2021 (21, 23, 28), 2022 (27, 35), 2023 (29, 30, 33, 34), and 2024 (31) (Table 1). Individual study sample sizes ranged from 20 to 103. Mean ages of participants across studies ranged from 47 to 66 years. Time post-stroke varied widely, ranging from 6 days to 8 months. Most participants were in the subacute (1–6 months post-stroke) or chronic phase (>6 months post-stroke) of stroke. Male predominance was observed in most studies. Dual-site TMS protocols with routine rehabilitation were applied in the experimental groups. Interventions included sham dual-site TMS with routine rehabilitation, single-site TMS with routine rehabilitation, or routine rehabilitation alone in the control groups. Routine rehabilitation comprised individualized physical and occupational therapy, including passive and active motor exercises, task-oriented training, and activities of daily living. The TMS parameters including stimulation site, frequency, intensity, and duration, varied in different individual studies (Table 1).

**Table 1:** Characteristic of included studies.

References (publication year)	Sample size	Age (year)	Gender (F/M)	Stroke type (I/H)	Time since stroke	Stroke phase	Affected hemisphere (L/R)	Intervention	TMS protocol	Outcomes
Long (2018) (20)	EG: 21	EG: 55.90 ± 8.89	EG: 5/16	EG: 11/10	EG: 19.81 ± 2.98 d	Acute	EG: 10/11	EG: rTMS+PT + OT	EG: cM1, 1 Hz rTMS, 90%RMT,1000 pulses, and iM1, 10 Hz rTMS, 90%RMT,1000 pulses, 5 times/weeks for 3 weeks	FMA-UL WMFT
	CG1: 20	CG1: 57 ± 11.78	CG1: 5/16	CG1: 11/10	CG1: 19.57 ± 2.34 d		CG1: 10/11	CG1: rTMS+PT + OT	CG1: cM1, 1 Hz rTMS, 90% RMT, 1000 pulses, 5 times/weeks for 3 weeks	
	CG2: 20	CG2: 56.85 ± 5.48	CG2: 5/15	CG2: 9/11	CG2: 19.05 ± 2.74 d		CG2: 9/11	CG2: sham rTMS+PT + OT	CG2: The coil was held at an angle of 90° to the scalp at the same sites in the same order as EG, 5 times/weeks for 3 weeks	
Cai (2020) (36)	EG: 52	EG: 55.37 ± 8.16	EG: 23/29	NR	EG: 60.31 ± 16.94 d	Subacute	EG: 26/26	EG: rTMS+PT + OT	EG: cM1, 1 Hz rTMS, 90% RMT, 1000 pulse, and iM1, 10 Hz rTMS, 90% RMT, 1000 pulses, 6 times/week for 4 weeks	FMA-UL, MBI, FTHUE-HK
	CG: 51	CG: 54.22 ± 7.59	CG: 21/30	NR	CG: 59.59 ± 15.93 d		CG: 22/29	CG: sham rTMS+PT + OT	CG: The coil was held at an angle of 90° to the scalp at the same sites in the same order as EG, 6 times/week for 4 weeks	
Sun (2024) (31)	EG: 10	EG: 53.8 ± 11.94	EG: 6/4	EG: 7/3	EG: 3–6 m	Subacute	EG: 4/6	EG: rTMS+PT + OT	EG: iM1, 10 Hz rTMS, 100%RMT, 1200 pulses, and cM1, 1 Hz rTMS, 100%RMT,1200 pulses, 6 times/week for 4 weeks	FMA-UL, MBI, ARAT
	CG: 10	CG: 48.6 ± 12.24	CG: 4/6	CG: 6/4	CG: 3–6 m		CG: 6/4	CG: rTMS+PT + OT	CG: iM1, 10 Hz rTMS, 100%RMT, 1200 pulses, 6 times/week for 4 weeks	
Chen (2021) (28)	EG: 25	EG: 58 (44.5, 65.5)	EG: 7/18	EG: 25/0	EG: 7 (5, 10) d	Acute	EG: 14/11	EG: rTMS+PT	EG: iM1, 10 Hz rTMS, 90%RMT, 600 pulses, and cM1, 1 Hz rTMS, 90%RMT,1200 pulses, 5 times/week for 4 weeks	FMA, FMA-UL, ADL, mRS,
	CG: 25	CG: 65 (52, 73)	CG: 7/18	CG: 25/0	CG: 5 (4, 9.5) d		CG: 12/13	CG: sham rTMS+PT	CG: sham rTMS, the gap between the coil and the cortex is substantially higher	
Cao (2022) (35)	EG: 20	EG: 51.80 ± 15.45	EG: 8/12	NR	EG: 45.85 ± 20.56 d	Subacute	EG: 13/7	EG: iTBS+PT + OT	EG: Controlesional cerebellar hemisphere, iTBS, 80-100%MT, 600 pulses, and iM1 iTBS, 80-100%MT, 600 pulses, 6 times/week for 4 weeks	FMA-UL, MBI, ARAT
	CG: 20	CG: 48.75 ± 13.24	CG: 9/11	NR	CG: 41.90 ± 24.86 d		CG: 8/12	CG: iTBS+PT + OT	CG: iM1 iTBS, 80-100%MT, 1200 pulses, 6 times/week for 4 weeks	
Ren (2018) (32)	EG: 30	EG: 51.2 ± 3.6	EG: 11/19	EG: 18/12	NR	NR	NR	EG: rTMS+PT + OT	EG: cM1, 1 Hz rTMS, 80% RMT, 5 minitues, and iM1, 5 Hz, 80% RMT, 15 min, 7 times/week for 2 weeks	FMA-UL, MBI
	CG: 30	CG: 49.6 ± 4.3	CG: 13/17	CG: 19/11	NR		NR	CG: rTMS+PT + OT	CG: iM1, 5 Hz, 80% RMT, 20 min, 7 times/week for 2 weeks	
Chen (2021) (21)	EG: 22	EG: 56 (39.3–64)	EG: 4/18	EG: 22/0	EG: 6 (3–9.5) d	Acute	EG: 9/13	EG: rTMS+PT	EG: iM1, 10 Hz rTMS, 90%RMT, 600 pulses, and cM1, 1 Hz rTMS, 90%RMT,1200 pulses, 5 times/week for 4 weeks	FMA, FMA-UL, BI, mRS
	CG: 22	CG: 60.5 (54.3–65.5)	CG: 7/15	CG: 22/0	CG: 6 (3–9.5) d		CG: 13/9	CG: sham rTMS+PT	CG: bilateral sham stimulation, the coil was placed on the skull surface in the reverse direction	
Chen (2022) (27)	EG: 16	EG: 53.25 (45.23, 61.27)	EG: 6/10	EG: 16/0	EG: 7.31 (5.29, 9.33) d	Acute	EG: 9/7	EG: rTMS+PT	EG: iM1, 10 Hz rTMS, 90%RMT, 600 pulses, and cM1, 1 Hz rTMS, 90%RMT,1200 pulses, 5 times/week for 4 weeks	FMA, FMA-UL, BI, mRS
	CG: 16	CG: 59.81 (54.41, 65.22)	CG: 4/12	CG: 16/0	CG: 7.94 (5.7, 10.18) d		CG: 6/10	CG: sham rTMS+PT	CG: bilateral sham stimulation, the coil was placed on the skull surface in the reverse direction	
Sung (2013) (22)	EG: 15	EG: 62.3 ± 12.2	EG: 3/12	EG: 10/5	EG: 7.8 ± 1.7 m	Chronic	NR	NR	EG: cM1, 1 Hz rTMS, 90%RMT, 600 pulses, and iM1, iTBS, 80% AMT, 600 pulses, 5 times/week for 4 weeks	FMA-UE, MRC, WMFT, FT, RT
	CG: 14	CG: 63.1 ± 12.8	CG: 3/11	CG: 9/5	CG: 8.2 ± 1.6 m		NR	NR	CG: a placebo coil for sham stimulation	
Huang (2023) (33)	EG: 18	EG: 53.44 ± 11.69	EG: 4/14	EG: 11/7	EG: 78.00 ± 29.28 d	Subacute	EG: 14/4	EG: rTMS+PT + OT	EG: cM1, 1 Hz rTMS, 90% RMT, 600 pulse, and iM1, 10 Hz rTMS, 90% RMT, 600 pulses, 5 times/week for 2 weeks	FMA-UE, MBI, MAS
	CG1: 18	CG1: 56.39 ± 10.57	CG1: 3/15	CG1: 11/7	CG1: 87.39 ± 37.48 d		CG1: 12/6	CG1: rTMS+PT + OT	CG1: iM1, 10 Hz rTMS, 90% RMT, 1200 pulses, 5 times/week for 2 weeks	
	CG2: 18	CG2: 52.94 ± 16.20	CG2: 6/12	CG2: 8/10	CG2: 65.5 ± 33.98 d		CG2: 9/9	CG2: sham rTMS+PT + OT	CG2: The coil was held at an angle of 90° to the scalp at the same sites in the same order as EG, 5 times/weeks for 2 weeks	
Xia (2023) (30)	EG1: 11	EG1: 47.18 ± 11.98	EG1: 1/10	EG1: 5/6	EG1: 50 (17) d	Subacute	EG1:6/5	EG1: iTBS	EG1: Controlesional cerebellar hemisphere, iTBS, 100%MT, 600 pulses, and iM1 iTBS, 100%MT, 600 pulses, 1 session	COP parameters
	EG2: 11	EG2: 50.36 ± 8.99	EG2: 1/10	EG2: 6/5	EG2: 50 (13) d		EG2: 5/6	EG2: iTBS	EG2: Controlesional cerebellar hemisphere, iTBS, 100%MT, 600 pulses, and ipsilateral SMA, 100%MT, 600 pulses, 1 session	
	CG: 9	CG: 54.44 ± 14.21	CG: 1/8	CG: 5/4	CG: 49 (25) d		CG: 5/4	CG: iTBS	CG: Controlesional cerebellar hemisphere, iTBS, 100%MT, 600 pulses, 1 session	
Chen (2023) (34)	EG: 30	EG: 65.8 ± 6.12	EG: 12/18	EG: 20/10	EG: 63.17 ± 18.33 d	Subacute	EG: 14/16	EG: iTBS+routine rehabilitation	EG: cM1, 1 Hz rTMS, 90%RMT, 1000 pulses, and iM1, iTBS, 80% RMT, 600 pulses, 5 times/week for 4 weeks	FMA-UE, MBI, MAS
	CG: 31	CG: 65.3 ± 7.02	CG: 15/16	CG: 23/8	CG: 63.58 ± 19.42 d		CG: 13/18	CG: routine rehabilitation	CG: no TMS	
Xue (2023) (29)	EG: 25	EG: 53.72 ± 6.79	EG: 13/12	EG: 14/11	EG: 33 ± 12.06 d	Subacute	NR	EG: rTMS+routine rehabilitation	EG: iM1, 5 Hz rTMS, 75%RMT, 600 pulses, and cM1, 1 Hz rTMS, 75%RMT,600 pulses, 5 times/week for 4 weeks	FMA-LE, MBI, BBS
	CG: 24	CG: 53.13 ± 4.12	CG: 9/15	CG: 12/12	CG: 36.63 ± 15.28 d		NR	CG: routine rehabilitation	CG: no TMS	
Li (2021) (23)	EG: 30	EG: 56.77 ± 8.58	EG: 10/20	EG: 24/6	EG: 3.63 ± 1.85 m	Subacute	NR	EG: TMS + routine rehabilitation	EG: cM1, 1 Hz rTMS, 80% RMT, 1000 pulses, and cerebellar hemisphere, cTBS, 80% AMT, 1200 pulses, 6 times/weeks for 4 weeks	MAS, FMA, BBS, MBI
	CG1: 30	CG1: 57.60 ± 7.4	CG1: 12/18	CG1: 23/7	CG1: 3.8 ± 1.71 m		NR	CG1: TMS + routine rehabilitation	CG1: cM1, 1 Hz rTMS, 80% RMT, 1000 pulses, 6 times/weeks for 4 weeks	
	CG2: 30	CG2: 55.13 ± 7.9	CG2: 11/19	CG2: 24/6	CG2: 3.67 ± 1.84 m		NR	CG2: TMS + routine rehabilitation	CG2: Cerebellar hemisphere, cTBS, 80% AMT, 1200 pulses, 6 times/weeks for 4 weeks	

EG = Experimental Group; CG = Control Group; SD = Standard Deviation; F/M = Femal/Male; I/H = Ischemic/hemorrhagic; TMS = Transcranial Magnetic Stimulation; m = month; w = week; d = day; iTBS = intermittent Theta Burst Stimulation; cTBS = continuous Theta Burst Stimulation; rTMS = repetitive Transcranial Magnetic Stimulation; M1 = primary motor cortex; LF = Low Frequency; PT = Physical Therapy; OT = Occupational Therapy; AMT = Active Motor Threshold; RMT = Resting Motor Threshold; 10MWT = 10 Meter Walking Test; TUG = Time Up and Go; BBS = Berg Balance Scale; BI = Barthel Index; MBI = Modified Barthel Index; ABC = Activity-specific Balance Confidence; SI = Stability Index; FMA = Fugl-Meyer Assessment; AE = Adverse Event; NR = Not Reported.

Outcome Measures focused on motor recovery and activities of daily living (ADL), assessed by Fugl-Meyer Assessment (FMA), FMA-Upper Limb (FMA-UL), Wolf Motor Function Test (WMFT), Action Research Arm Test (ARAT), Modified Barthel Index (MBI), Barthel Index (BI), Modified Ashworth Scale (MAS), Berg Balance Scale (BBS), and others.

### Methodological quality

According to PEDro scores (Table 2), thirteen studies were rated as high quality, and one as moderate quality. All studies reported comparable baseline characteristics between groups, between-group comparisons, and provided point estimates along with measures of variability. However, most studies did not meet the criteria for items related to participant blinding (item 5), therapist blinding (item 6), and concealed allocation (item 3), reflecting the methodological challenges commonly encountered in rehabilitation trials. Additionally, some studies lacked assessor blinding (item 7) and intention-to-treat analysis (item 9), which may have introduced bias into the results and affected the internal validity of the studies.

**Table 2:** PEDro score for methodological quality assessment of including studies.

Reference	Item 1	Item 2	Item 3	Item 4	Item 5	Item 6	Item 7	Item 8	Item 9	Item 10	Item 11	Score
Long (2018) (20)	1	1	0	1	0	0	1	1	1	1	1	7
Cai (2020) (36)	1	1	0	1	0	0	1	1	0	1	1	6
Sun (2024) (31)	1	1	0	1	0	0	1	1	1	1	1	7
Chen (2021) (28)	1	1	1	1	1	1	0	0	0	1	1	7
Cao (2022) (35)	1	1	0	1	0	0	1	1	1	1	1	7
Ren (2018) (32)	1	1	0	1	0	0	0	1	0	1	1	5
Chen (2021) (21)	1	1	1	1	1	0	1	1	0	1	1	8
Chen (2022) (27)	1	1	1	1	1	1	1	1	0	1	1	9
Sung (2013) (22)	1	1	1	1	0	1	1	1	1	1	1	9
Huang (2023) (33)	1	1	1	1	0	1	1	1	0	1	1	8
Xia (2023) (30)	1	1	1	1	1	0	0	1	1	1	1	8
Chen (2023) (34)	1	1	0	1	0	0	1	1	0	1	1	6
Xue (2023) (29)	1	1	0	1	0	0	1	1	1	1	1	7
Li (2021) (23)	1	1	0	1	0	0	0	1	1	1	1	6

Item 1 = eligibility criteria, Item 2 = random allocation, Item 3 = concealed allocation, Item 4 = similar baseline, Item 5 = subjected blinded, Item 6 = therapists blinded, Item 7 = assessors blinded, Item 8 = < 15% dropouts, Item 9 = intention-to-treat analysis, Item 10 = between-group comparison, Item 11 = point measures and variability data, 1 = described explicitly and in details, 0 = unclear, inadequately described.

### Outcome measures

#### FMA-UL

**Figure 2:**
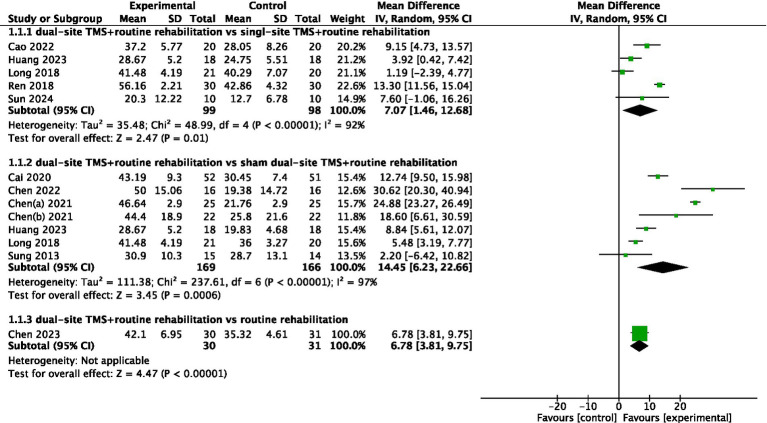
Forest plot for FMA-UL.

A total of eleven studies reported FMA-UL results. The pooled meta-analysis demonstrated that dual-site TMS combined with routine rehabilitation was significantly more effective than single-site TMS with routine rehabilitation in improving FMA-UL scores (MD, 7.07; 95% CI, 1.46 to 12.68; I^2^ = 92%, *p* < 0.001; Figure 2). Subgroup analyses indicated that patients in the subacute phase showed significant improvements in the experimental group (Table 3). Additionally, both treatment sessions ≤20 and >20 resulted in significant FMA-UL improvements in the experimental group (Table 3). When compared to sham stimulation with routine rehabilitation, dual-site TMS with routine rehabilitation also showed significant FMA-UL improvement (MD, 14.45; 95% CI, 6.23 to 22.66; I^2^ = 97%, *p* < 0.001; Figure 2). Subgroup analyses revealed that both acute and subacute patients experienced significant FMA-UL improvements in the experimental group, regardless of whether treatment sessions were ≤20 or >20 (Table 3). Only one study (34) reported that dual-site TMS with routine rehabilitation was significantly more effective than routine rehabilitation alone in improving FMA-UL scores (Figure 2).

**Table 3:** Overall and subgroup analysis.

Outcomes	Comparison	Overall and subgroup analysis	No. of study	No. of participant	MD (95% CI)	*p*-value	Heterogeneity		
							Chi^2^	*p*-value	I^2^
FMA-UL	Comparison I	Overall	5	197	7.07 (1.46, 12.68)	0.01	48.99	<0.00001	92%
		Post-stroke duration (<1 month)	1	41	1.19 (−2.39, 4.77)	0.51	/	/	/
		Post-stroke duration (1–3 month)	3	96	6.46 (2.71, 10.21)	0.0007	3.44	0.18	42%
		Post-stroke duration (>6 month)	0	/	/	/	/	/	/
		Treatment sessions (≤20)	3	137	6.25 (−1.96, 14.47)	0.14	48.68	<0.00001	96%
		Treatment sessions (>20)	2	60	8.83 (4.9, 12.76)	<0.0001	0.1	0.75	0%
	Comparison II	Overall	7	335	14.45 (6.23, 22.66)	0.0006	237.61	<0.00001	97%
		Post-stroke duration (<1 month)	4	167	18.69 (17.39, 19.98)	<0.00001	189.4	<0.00001	98%
		Post-stroke duration (1–3 month)	2	139	10.78 (8.49, 13.07)	<0.00001	2.79	0.09	64%
		Post-stroke duration (>6 month)	1	29	2.2 (−6.42, 10.82)	0.62	/	/	/
		Treatment sessions (≤20)	6	232	17.03 (15.83, 18.22)	<0.00001	231.69	<0.00001	98%
		Treatment sessions (>20)	1	103	12.74 (9.5, 15.98)	<0.00001	/	/	/
ADL	Comparison I	Overall	6	246	9.9 (7.82, 11.98)	<0.00001	5.22	0.39	4%
		Post-stroke duration (<1 month)	0	/	/	/	/	/	/
		Post-stroke duration (1–3 month)	4	186	6.54 (2.26, 10.83)	0.003	1.87	0.76	0%
		Post-stroke duration (>6 month)	0	/	/	/	/	/	/
		Treatment sessions (≤20)	2	96	10.71 (8.91, 12.52)	<0.00001	1.17	0.28	14%
		Treatment sessions (>20)	3	150	6.82 (2.06, 11.57)	0.005	1.8	0.61	0%
	Comparison II	Overall	5	265	21.13 (9.37, 32.88)	0.0004	187.28	<0.00001	98%
		Post-stroke duration (<1 month)	3	126	29.62 (27.9, 21.34)	<0.00001	3.24	0.2	38%
		Post-stroke duration (1–3 month)	2	139	11.17 (9.13, 13.22)	<0.00001	0.98	0.32	0%
		Post-stroke duration (>6 month)	0	/	/	/	/	/	/
		Treatment sessions (≤20)	4	162	29.25 (27.56, 30.95)	<0.00001	9.68	0.02	69%
		Treatment sessions (>20)	1	103	10.96 (8.87, 13.05)	<0.00001	/	/	/
	Comparison III	Overall	2	110	7.84 (−1.24, 16.93)	0.09	8.09	0.004	88%
		Post-stroke duration (<1 month)	0	/	/	/	/	/	/
		Post-stroke duration (1–3 month)	2	110	7.84 (−1.24, 16.93)	0.09	8.09	0.004	88%
		Post-stroke duration (>6 month)	0	/	/	/	/	/	/
		Treatment sessions (≤20)	2	110	7.84 (−1.24, 16.93)	0.09	8.09	0.004	88%
		Treatment sessions (>20)	0	/	/	/	/	/	/
FMA	Comparison I	Overall	1	90	11.42 (5.6, 17.25)	0.0001	/	/	/
	Comparison II	Overall	3	126	35.15 (26.86, 43.44)	<0.00001	1.81	0.4	0%
		Post-stroke duration (<1 month)	3	126	35.15 (26.86, 43.44)	<0.00001	1.81	0.4	0%
		Post-stroke duration (1–3 month)	0	/	/	/	/	/	/
		Post-stroke duration (>6 month)	0	/	/	/	/	/	/
		Treatment sessions (≤20)	3	126	35.15 (26.86, 43.44)	<0.00001	1.81	0.4	0%
		Treatment sessions (>20)	0	/	/	/	/	/	/
WMFT	Comparison I	Overall	1	41	−17.38 (−25.15, −9.61)	<0.00001	/	/	/
	Comparison II	Overall	2	70	−8.26 (−37.52, 21.01)	0.58	20.8	<0.00001	95%
		Post-stroke duration (<1 month)	1	41	−22.87 (−29.59, −16.15)	<0.00001	/	/	/
		Post-stroke duration (1–3 month)	0	/	/	/	/	/	/
		Post-stroke duration (>6 month)	1	29	9 (−3.94, 17.94)	0.21	/	/	/
		Treatment sessions (≤20)	2	70	−8.26 (−37.52, 21.01)	0.58	20.8	<0.00001	95%
		Treatment sessions (>20)	0	/	/	/	/	/	/
ARAT	Comparison I	Overall	2	60	4.9 (2.35, 7.45)	0.0002	5.29	0.02	81%
		Post-stroke duration (<1 month)	0	/	/	/	/	/	/
		Post-stroke duration (1–3 month)	2	60	4.9 (2.35, 7.45)	0.0002	5.29	0.02	81%
		Post-stroke duration (>6 month)	0	/	/	/	/	/	/
		Treatment sessions (≤20)	0	/	/	/	/	/	/
		Treatment sessions (>20)	2	60	4.9 (2.35, 7.45)	0.0002	5.29	0.02	81%
	Comparison II	Overall	3	126	−1.37 (−1.99, −0.75)	<0.00001	16.86	0.0002	88%
		Post-stroke duration (<1 month)	3	126	−1.37 (−1.99, −0.75)	<0.00001	16.86	0.0002	88%
		Post-stroke duration (1–3 month)	0	/	/	/	/	/	/
		Post-stroke duration (>6 month)	0	/	/	/	/	/	/
		Treatment sessions (≤20)	3	126	−1.37 (−1.99, −0.75)	<0.00001	16.86	0.0002	88%
		Treatment sessions (>20)	0	/	/	/	/	/	/
MAS	Comparison I	Overall	2	126	−0.7 (−2.3, 0.91)	0.39	13.82	0.001	86%
		Post-stroke duration (<1 month)	0	/	/	/	/	/	/
		Post-stroke duration (1–3 month)	2	126	−0.7 (−2.3, 0.91)	0.39	13.82	0.001	86%
		Post-stroke duration (>6 month)	0	/	/	/	/	/	/
		Treatment sessions (≤20)	1	36	1 (−0.19, 2,19)	0.1	/	/	/
		Treatment sessions (>20)	1	90	−1.5 (−2.2, −0.8)	<0.00001	/	/	/
	Comparison II	Overall	1	36	0 (−1.33, 1.33)	1	/	/	/
	Comparison III	Overall	1	61	−0.59 (−0.99, −0.18)	0.004	/	/	/

Comparison I: dual-site TMS + routine rehabilitation vs single-site TMS + routine rehabilitation; Comparison II: dual-site TMS + routine rehabilitation vs sham dual-site TMS + routine rehabilitation; Comparison III: dual-site TMS + routine rehabilitation vs routine rehabilitation.

#### ADL

**Figure 3:**
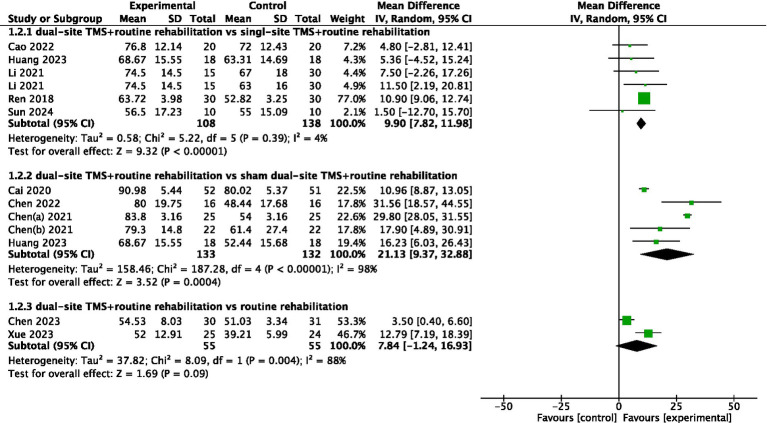
Forest plot for ADL.

Eleven studies reported ADL-related outcomes, with two using the BI and nine using the MBI. The pooled meta-analysis indicated that dual-site TMS with routine rehabilitation was significantly more effective than single-site TMS with routine rehabilitation in improving ADL (MD, 9.90; 95% CI, 7.82 to 11.98; I^2^ = 4%, *p* = 0.39; Figure 3). Subgroup analyses showed that both treatment sessions ≤20 and >20 led to significant ADL improvements in the experimental group (Table 3). Compared to sham stimulation with routine rehabilitation, dual-site TMS with routine rehabilitation was significantly more effective in ADL (MD, 21.13; 95% CI, 9.37 to 32.88; I^2^ = 98%, *p* < 0.001; Figure 3). Subgroup analyses again showed significant improvements in both acute and subacute patients, regardless of treatment session number (Table 3). However, a pooled meta-analysis of two studies found that dual-site TMS with routine rehabilitation was not significantly more effective than routine rehabilitation alone in ADL (MD, 7.84; 95% CI, −1.24 to 16.93; I^2^ = 88%, *p* = 0.004; Figure 3).

#### FMA

**Figure 4:**
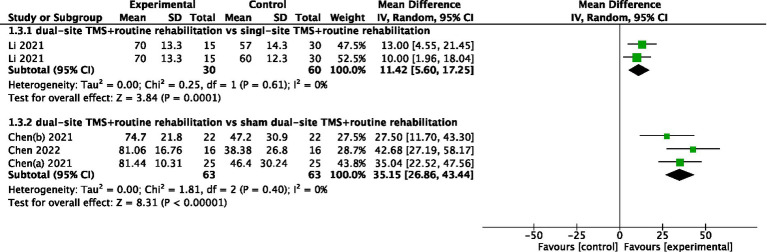
Forest plot for FMA.

Four studies reported FMA results. The pooled meta-analysis showed that dual-site TMS with routine rehabilitation was significantly more effective than single-site TMS with routine rehabilitation (MD, 11.42; 95% CI, 5.60 to 17.25; I^2^ = 0%, *p* = 0.61; Figure 4). Similarly, dual-site TMS with routine rehabilitation was significantly more effective than sham stimulation with routine rehabilitation (MD, 35.15; 95% CI, 26.86 to 43.44; I^2^ = 0%, *p* = 0.40; Figure 4).

#### WMFT

**Figure 5:**
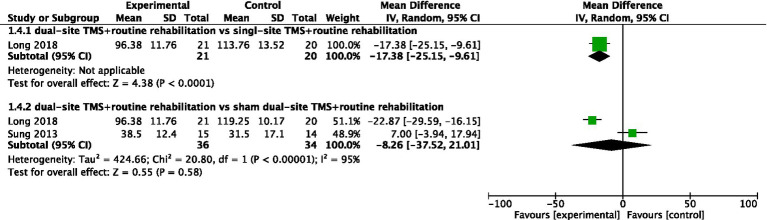
Forest plot for WMFT.

Two studies reported WMFT results. While one study found dual-site TMS with routine rehabilitation to be significantly more effective than single-site TMS with routine rehabilitation, the pooled meta-analysis of two studies showed no significant difference between dual-site TMS with routine rehabilitation and sham stimulation with routine rehabilitation (MD, −8.26; 95% CI, −37.52 to 21.01; I^2^ = 95%, *p* < 0.001; Figure 5).

#### ARAT

**Figure 6:**
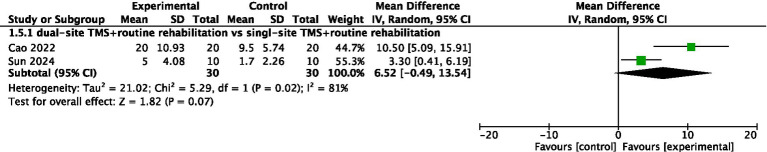
Forest plot for ARAT.

Two studies reported ARAT results. The pooled meta-analysis indicated no significant difference between dual-site TMS with routine rehabilitation and single-site TMS with routine rehabilitation (MD, 6.52; 95% CI, −0.49 to 13.54; I^2^ = 81%, *p* = 0.02; Figure 6).

#### mRS

**Figure 7:**
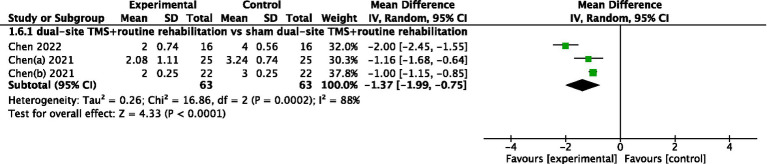
Forest plot for mRS.

Three studies reported mRS results. The pooled meta-analysis demonstrated that dual-site TMS with routine rehabilitation was significantly more effective than sham stimulation with routine rehabilitation (MD, −1.37; 95% CI, −1.99 to −0.75; I^2^ = 88%, *p* < 0.001; Figure 7).

#### MAS

**Figure 8:**
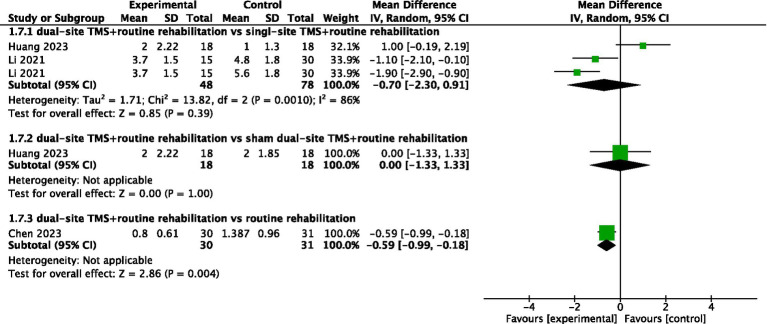
Forest plot for MAS.

Three studies reported MAS results. The pooled meta-analysis showed no significant difference between dual-site TMS with routine rehabilitation and single-site TMS with routine rehabilitation (MD, −0.70; 95% CI, −2.30 to 0.91; I^2^ = 86%, *p* = 0.001; Figure 8). However, subgroup analyses indicated that treatment sessions >20 achieved significant MAS improvements in the experimental group (Table 3). One study (33) found no significant difference between dual-site TMS with routine rehabilitation and sham stimulation with routine rehabilitation in MAS. Another study (34) reported that dual-site TMS with routine rehabilitation was significantly more effective than routine rehabilitation alone in MAS.

### Other outcomes

One study (29) showed that dual-site TMS with routine rehabilitation was significantly more effective than routine rehabilitation alone in FMA-LL. One study (36) showed that dual-site TMS with routine rehabilitation was significantly more effective than single-site TMS with routine rehabilitation in FTHUE-HK. One study (22) showed that dual-site TMS with routine rehabilitation was significantly more effective than sham TMS with routine rehabilitation in finger flexor MRC, simple reaction time task, and index finger tapping task. Another study (30) demonstrated that a single session of dual-site iTBS could immediately improve balance function in patients with eyes closed compared to single-site iTBS.

### Sensitivity analysis and publication bias

Leave-one-out sensitivity analyses indicated that sequentially excluding each included study did not significantly alter the overall effect estimates, with most results remaining within the 95% confidence intervals. Notably, when excluding Cao et al. (35), the pooled meta-analysis showed that dual-site TMS with routine rehabilitation was not significantly more effective than single-site TMS with routine rehabilitation in FMA-UL (MD, 6.53; 95% CI, −0.51 to 13.56; I^2^ = 96%, *p* < 0.00001). These findings suggest that most meta-analysis results were robust and not unduly influenced by any single study. Publication bias was not formally assessed, as individual meta-analyses did not include 10 or more studies.

### Adverse events and side effects

All participants tolerated DS-NIBS without significant adverse events. Four of the studies mentioned that subjects experienced transient mild headaches and slight tingling sensations during TMS stimulation (21, 27, 28, 36). However, the symptoms were well-tolerated and resolved immediately after treatment, and did not interfere with subsequent therapeutic sessions.

### Quality of evidence

For FMA-UL, the evidence quality was low when comparing dual-site TMS combined with routine rehabilitation to single-site TMS with routine rehabilitation. However, it reaches moderate quality when compared to sham dual-site TMS with routine rehabilitation, and it drops to very low quality when compared to routine rehabilitation alone. This variation is influenced by factors such as serious inconsistency in some comparisons and extremely serious imprecision in others.

Regarding ADL, the evidence quality was low when dual-site TMS with routine rehabilitation is compared to single-site TMS with routine rehabilitation. It falls to very low quality when compared to both sham dual-site TMS with routine rehabilitation and routine rehabilitation alone. The main issues here include serious inconsistency and very serious imprecision.

For other outcomes, the evidence quality was very low across all comparisons, whether it’s with single-site TMS, sham dual-site TMS, or routine rehabilitation. Detailed ratings and justifications are provided in the Supplementary Table 2.

## Discussion

This systematic review and meta-analysis provides compelling evidence for the effectiveness of dual-site transcranial magnetic stimulation (TMS) in enhancing motor function and activities of daily living (ADL) in stroke patients. Across 14 randomized controlled trials with 724 participants, dual-site TMS demonstrated significant advantages over single-site TMS and sham stimulation. Notably, improvements in upper limb motor function (MD, 7.07; 95% CI, 1.46 to 12.68; *p* < 0.001) and ADL (MD, 9.90; 95% CI, 7.82 to 11.98; *p* = 0.39) highlight its potential as a valuable adjunct to conventional rehabilitation therapies.

Dual-site TMS is an innovative neuromodulation technique used in stroke rehabilitation that targets multiple brain regions in close succession. This approach enhances understanding of brain network interactions and has shown potential to improve motor recovery in stroke survivors (37, 38). The efficacy of dual-site TMS could be attributed to its unique ability to modulate neural activity across distributed brain networks disrupted by stroke (39, 40). By sequentially stimulating two distinct brain regions, such as bilateral M1 or M1 and the cerebellum, dual-site TMS promotes synchronized neural oscillations and enhances interregional connectivity (41). This dual stimulation facilitates rebalancing of interhemispheric inhibition, which is often disrupted post-stroke, and activates diaschisis-related pathways that become inhibited secondary to the primary lesion (42, 43). Preclinical studies indicate that dual-site stimulation may induce more robust long-term potentiation (LTP) effects compared to single-site protocols, suggesting stronger neuroplastic changes underlying motor recovery (7).

A resting-state fMRI study revealed that rTMS increased functional activity and connectivity in motor-related brain regions for stroke patients, highlighting rTMS’s role in modulating neural networks to support motor rehabilitation (44). Stroke patients exhibit individualized cortical responses to rTMS, and that tailoring rTMS neuromodulation significantly improves motor imagery decoding and functional recovery (45). Cerebellar TMS enhanced balance and lower limb motor function in stroke patients, spontaneous neural activity alterations were identified in motor-related regions after stroke, including the precentral gyrus, putamen, thalamus, and paracentral lobule based on fMRI studies (46). Another systematic review of fMRI studies demonstrated that the neural mechanism of rTMS in improving motor function after stroke may be the activation and functional connectivity of motor-related brain areas, including enhancement of the activation of motor-related brain areas in the affected hemisphere, inhibition of the activation of motor-related brain areas in the unaffected hemisphere, and changing the functional connectivity of intra-hemispheric and inter-hemispheric motor networks (47).

Subgroup analyses indicated that dual-site TMS was especially effective during the acute and subacute phases of stroke recovery, with significant improvements in FMA-UL scores. This observation is consistent with the enhanced neuroplastic potential of the brain during the early post-stroke period, as supported by animal and human studies (48–50). Early intervention may therefore facilitate greater reorganization of motor pathways (51). Additionally, treatment outcomes were influenced by the number of TMS sessions. Both ≤20 and >20 sessions yielded significant functional gains; however, the >20 sessions subgroup demonstrated greater mean improvements. This finding supports the dose–response relationship of cumulative neuromodulatory effects on cortical excitability and synaptic plasticity (7, 52). These results underscore the importance of treatment duration and intensity in optimizing clinical efficacy”.

The frequency of rTMS plays a crucial role in modulating cortical excitability. Specifically, high-frequency rTMS (≥5 Hz) is generally excitatory and facilitates cortical activity, particularly over the ipsilesional motor cortex (M1), while low-frequency rTMS (≤1 Hz) exerts inhibitory effects, typically applied to the contralesional hemisphere to suppress maladaptive interhemispheric inhibition (7, 9). Dual-site TMS protocols strategically combine these two modalities to rebalance interhemispheric interactions—enhancing neural excitability in the affected hemisphere while concurrently reducing overactivity in the unaffected hemisphere. For instance, Long et al. (20) and Cai et al. (36) applied this dual-frequency strategy and reported significant improvements in upper-limb motor function in subacute stroke patients.

Stimulation intensity, typically expressed as a percentage of the RMT, plays a crucial role in treatment efficacy (53, 54). Higher intensities may produce more pronounced neurophysiological effects but also increase adverse event risks (55). Most studies used intensities ranging from 80 to 100% RMT without serious adverse effects. The duration of TMS sessions and overall treatment period also influence outcomes. Longer treatment durations may allow for more sustained neuroplastic changes (52). However, studies varied in their approaches, with treatment periods ranging from 2 to 4 weeks. Further research is needed to establish the ideal duration for dual-site TMS interventions.

Our findings corroborate and extend previous research on TMS in stroke rehabilitation. Earlier studies (56, 57) showed modest effects of single-site TMS on motor function and cognitive function, but dual-site TMS appears to offer enhanced benefits (58). For instance, a meta-analysis (9) found moderate effects of single-site rTMS on upper limb function, while our results indicate that dual-site TMS may provide superior outcomes. Additionally, another systematic review (16) reported that dual-site non-invasive brain stimulation outperformed single-site non-invasive brain stimulation in improving upper limb function and ADL.

Integrating dual-site TMS into clinical practice shows significant promise but presents challenges. Current guidelines from the American Heart Association/American Stroke Association (AHA/ASA) recognize TMS as a potential adjunct to stroke rehabilitation, but evidence for dual-site TMS is still emerging (3). Our findings support considering dual-site TMS in clinical settings, particularly for subacute patients. However, standardized protocols and cost-effectiveness evaluations are needed before widespread adoption. Variability in the parameters of rTMS, including stimulation protocols, locations of stimulation, and frequency, further complicates the establishment of standardized treatment guidelines.

The dual-site TMS protocol in our meta-analysis involved stimulation over the ipsilesional M1 as at least one of the target sites, often in conjunction with either the contralateral M1 or the cerebellum. This variability may have contributed to some heterogeneity in treatment effects and should be more rigorously standardized in future trials. The M1 is the most frequently targeted region in post-stroke neuromodulation research due to its central role in initiating voluntary movement via the corticospinal tract (8, 57). Modulating excitability in the lesioned M1 (via high-frequency rTMS) or/and downregulating the contralesional M1 (via low-frequency rTMS) could restore interhemispheric balance and improve motor outcomes (8, 57).

Future research should prioritize more head-to-head comparisons of dual-site TMS protocols to determine optimal stimulation parameters and target regions. Long-term follow-up studies are necessary to assess durability of treatment effects. Incorporating advanced neuroimaging techniques like fMRI and DTI can provide insights into neural mechanisms and guide personalized treatment approaches. Additionally, exploring dual-site TMS in combination with other therapies, such as brain-computer interfaces (BCIs) or pharmacological agents, may further enhance rehabilitation outcomes.

Several limitations should be considered when interpreting our results. One limitation of this review is the absence of a prospectively registered protocol, which may raise concerns about selective reporting or methodological bias. Only 14 RCTs met our inclusion criteria, underscoring the need for larger, multi-center trials to confirm these findings. Heterogeneity among studies in participant characteristics, TMS protocols, and outcome measures may have influenced pooled effect estimates. Most studies did not adequately address participant or therapist blinding, potentially introducing biases. Short follow-up periods limit our ability to assess long-term efficacy.

Another major limitation of the studies included in our analysis is the homogeneity of the sample population, all participants were recruited in Chinese population, limiting generalizability to other ethnic and healthcare contexts. This raises concerns about the generalizability of the findings to other racial and ethnic groups. Future multi-center trials across diverse populations are warranted.

## Conclusion

This systematic review and meta-analysis demonstrates that dual-site TMS combined with routine rehabilitation is effective in improving motor function and ADL in stroke patients. The findings highlight its potential to enhance neuroplasticity and functional recovery through dual-target stimulation. While the results are encouraging, further research is needed to optimize treatment protocols and address methodological limitations. Further research is required to establish the efficacy of dual-site TMS in larger, multicenter trials and to optimize treatment protocols, thereby ensuring robust evidence supporting its integration into standard rehabilitation practices.

## Data Availability

The original contributions presented in the study are included in the article/Supplementary material, further inquiries can be directed to the corresponding authors.

## Footnotes

^1^ http://getdata-graph-digitizer.com/
