# Supplementary material for: Effectiveness of dual-site transcranial magnetic stimulation on motor function and activities of daily living in stroke patients: a systematic review and meta-analysis of randomized controlled trials
Source: Front Neurol. 2025 Jul 14;16:1630876. doi: 10.3389/fneur.2025.1630876 (PMC12301190; doi:10.3389/fneur.2025.1630876)
Supplement: Supplementary file 1 [file Table_1.docx]

Supplement Table 1. Search strategy in PubMed

| #1 | "Transcranial Magnetic Stimulation"[MeSH Terms] OR "magnetic stimulation transcranial"[Title/Abstract] OR "stimulation transcranial magnetic"[Title/Abstract] OR "Transcranial Magnetic Stimulations"[Title/Abstract] OR "theta burst stimulation"[Title/Abstract] OR "iTBS"[Title/Abstract] OR "cTBS"[Title/Abstract] OR "TMS"[Title/Abstract] OR "rTMS"[Title/Abstract] |
| --- | --- |
| #2 | "Stroke"[MeSH Terms] OR "Stroke"[Title/Abstract] OR "cerebrovascular accident*"[Title/Abstract] OR "cva"[Title/Abstract] OR "cerebrovascular apoplexy"[Title/Abstract] OR "brain vascular accident*"[Title/Abstract] OR "cerebrovascular stroke*"[Title/Abstract] OR "cerebral stroke*"[Title/Abstract] OR "cerebrovascular accident*"[Title/Abstract] |
| #3 | "motor skills"[MeSH Terms] OR "motor disorders"[MeSH Terms] OR "motor activity"[MeSH Terms] OR "movement"[MeSH Terms] OR "muscle strength"[MeSH Terms] OR "gait"[MeSH Terms] OR "postural balance"[MeSH Terms] OR "muscle spasticity"[MeSH Terms] OR "functional status"[MeSH Terms] OR "activities of daily living"[MeSH Terms] OR "motor"[Title/Abstract] OR "strength"[Title/Abstract] OR "gait"[Title/Abstract] OR "Postural"[Title/Abstract] OR "Balance"[Title/Abstract] OR "spasticity"[Title/Abstract] OR "walking"[Title/Abstract] OR "upper limb"[Title/Abstract] OR "lower limb"[Title/Abstract] OR "activities of daily living"[Title/Abstract] OR "ADL"[Title/Abstract] |
| #4 | ("randomized controlled trial"[Publication Type] OR "controlled clinical trial"[Publication Type] OR "randomized"[Title/Abstract] OR "placebo"[Title/Abstract] OR "clinical trials as topic"[MeSH Terms:noexp] OR "randomly"[Title/Abstract] OR "trial"[Title]) |
| #5 | #1 AND #2 AND #3 AND #4 |
