# Supplementary material for: Effectiveness of dual-site transcranial magnetic stimulation on motor function and activities of daily living in stroke patients: a systematic review and meta-analysis of randomized controlled trials
Source: Front Neurol. 2025 Jul 14;16:1630876. doi: 10.3389/fneur.2025.1630876 (PMC12301190; doi:10.3389/fneur.2025.1630876)
Supplement: Supplementary file 2 [file Table_2.docx]

Supplement Table 2. Summary of quality of evidence for outcomes

| **Certainty assessment** | | | | | | | **№ of patients** | | **Effect** | | **Certainty** | **Importance** |
| --- | --- | --- | --- | --- | --- | --- | --- | --- | --- | --- | --- | --- |
| **№ of studies** | **Study design** | **Risk of bias** | **Inconsistency** | **Indirectness** | **Imprecision** | **Other considerations** | **dual-site TMS** | **single-site TMS or sham dual-site TMS** | **Relative (95% CI)** | **Absolute (95% CI)** |  |  |
| **FMA-UL - dual-site TMS+routine rehabilitation vs singl-site TMS+routine rehabilitation** | | | | | | | | | | | | |
| 5 | randomised trials | not serious | serious^a^ | not serious | serious^b^ | none | 99 | 98 | - | MD **7.07 higher** (1.46 higher to 12.68 higher) | ⨁⨁◯◯ Low | CRITICAL |
| **FMA-UL - dual-site TMS+routine rehabilitation vs sham dual-site TMS+routine rehabilitation** | | | | | | | | | | | | |
| 7 | randomised trials | not serious | serious^a^ | not serious | not serious | none | 169 | 166 | - | MD **14.45 higher** (6.23 higher to 22.66 higher) | ⨁⨁⨁◯ Moderate | CRITICAL |
| **FMA-UL - dual-site TMS+routine rehabilitation vs routine rehabilitation** | | | | | | | | | | | | |
| 1 | randomised trials | not serious | serious^c^ | not serious | extremely serious^d^ | none | 30 | 31 | - | MD **6.78 higher** (3.81 higher to 9.75 higher) | ⨁◯◯◯ Very low | CRITICAL |
| **ADL - dual-site TMS+routine rehabilitation vs singl-site TMS+routine rehabilitation** | | | | | | | | | | | | |
| 5 | randomised trials | not serious | not serious | not serious | very serious^b^ | none | 108 | 138 | - | MD **9.9 higher** (7.82 higher to 11.98 higher) | ⨁⨁◯◯ Low | CRITICAL |
| **ADL - dual-site TMS+routine rehabilitation vs sham dual-site TMS+routine rehabilitation** | | | | | | | | | | | | |
| 5 | randomised trials | not serious | serious^a^ | not serious | very serious^b^ | none | 133 | 132 | - | MD **21.13 higher** (9.37 higher to 32.88 higher) | ⨁◯◯◯ Very low | CRITICAL |
| **ADL - dual-site TMS+routine rehabilitation vs routine rehabilitation** | | | | | | | | | | | | |
| 2 | randomised trials | not serious | serious^e^ | not serious | extremely serious^d,e^ | none | 55 | 55 | - | MD **7.84 higher** (1.24 lower to 16.93 higher) | ⨁◯◯◯ Very low | CRITICAL |
| **FMA - dual-site TMS+routine rehabilitation vs singl-site TMS+routine rehabilitation** | | | | | | | | | | | | |
| 1 | randomised trials | not serious | serious^c^ | not serious | extremely serious^c^ | none | 30 | 60 | - | MD **11.42 higher** (5.6 higher to 17.25 higher) | ⨁◯◯◯ Very low | IMPORTANT |
| **FMA - dual-site TMS+routine rehabilitation vs sham dual-site TMS+routine rehabilitation** | | | | | | | | | | | | |
| 3 | randomised trials | not serious | serious^a^ | not serious | extremely serious^c^ | none | 63 | 63 | - | MD **35.15 higher** (26.86 higher to 43.44 higher) | ⨁◯◯◯ Very low | IMPORTANT |
| **WMFT - dual-site TMS+routine rehabilitation vs singl-site TMS+routine rehabilitation** | | | | | | | | | | | | |
| 1 | randomised trials | not serious | serious^c^ | not serious | extremely serious^c^ | none | 21 | 20 | - | MD **17.38 lower** (25.15 lower to 9.61 lower) | ⨁◯◯◯ Very low | IMPORTANT |
| **WMFT - dual-site TMS+routine rehabilitation vs sham dual-site TMS+routine rehabilitation** | | | | | | | | | | | | |
| 2 | randomised trials | not serious | serious^a^ | not serious | extremely serious^d,e^ | none | 36 | 34 | - | MD **8.26 lower** (37.52 lower to 21.01 higher) | ⨁◯◯◯ Very low | IMPORTANT |
| **ARAT - dual-site TMS+routine rehabilitation vs singl-site TMS+routine rehabilitation** | | | | | | | | | | | | |
| 2 | randomised trials | not serious | serious^a^ | not serious | extremely serious^d,e^ | none | 30 | 30 | - | MD **6.52 higher** (0.49 lower to 13.54 higher) | ⨁◯◯◯ Very low | IMPORTANT |
| **mRS - dual-site TMS+routine rehabilitation vs sham dual-site TMS+routine rehabilitation** | | | | | | | | | | | | |
| 3 | randomised trials | not serious | serious^a^ | not serious | very serious^b^ | none | 63 | 63 | - | MD **1.37 lower** (1.99 lower to 0.75 lower) | ⨁◯◯◯ Very low | IMPORTANT |
| **MAS - dual-site TMS+routine rehabilitation vs singl-site TMS+routine rehabilitation** | | | | | | | | | | | | |
| 2 | randomised trials | not serious | serious^a^ | not serious | very serious^b,e^ | none | 48 | 78 | - | MD **0.7 lower** (2.3 lower to 0.91 higher) | ⨁◯◯◯ Very low | IMPORTANT |
| **MAS - dual-site TMS+routine rehabilitation vs sham dual-site TMS+routine rehabilitation** | | | | | | | | | | | | |
| 1 | randomised trials | not serious | serious^c^ | not serious | extremely serious^c,e^ | none | 18 | 18 | - | MD **0**  (1.33 lower to 1.33 higher) | ⨁◯◯◯ Very low | IMPORTANT |
| **MAS - dual-site TMS+routine rehabilitation vs routine rehabilitation** | | | | | | | | | | | | |
| 1 | randomised trials | not serious | serious^c^ | not serious | extremely serious^c^ | none | 30 | 31 | - | MD **0.59 lower** (0.99 lower to 0.18 lower) | ⨁◯◯◯ Very low | IMPORTANT |
| **FMA-LL - dual-site TMS+routine rehabilitation vs routine rehabilitation** | | | | | | | | | | | | |
| 1 | randomised trials | not serious | serious^c^ | not serious | extremely serious^c^ | none | 25 | 24 | - | MD **6.22 higher** (4.34 higher to 8.1 higher) | ⨁◯◯◯ Very low | IMPORTANT |

CI: confidence interval; MD: mean difference

GRADE Working Group grades of evidence

High quality: Further research is very unlikely to change our confidence in the estimate of effect

Moderate quality: Further research is likely to have an important impact on our confidence in the estimate of effect and may change the estimate

Low quality: Further research is very likely to have an important impact on our confidence in the estimate of effect and is likely to change the estimate

Very low quality: We are very uncertain about the estimate

a. The included studies showed high heterogeneity (I^2^>90% or *P*<0.05)

b. Sample size was relatively small (<300)

c. Only one included study

d. Sample size was very small (<100)

e. Effect direction was inconsistent
